# Interaction of dietary patterns with rs28362491 on severity of coronary artery stenosis in patients undergoing coronary angiography

**DOI:** 10.1038/s41598-023-41438-1

**Published:** 2023-09-05

**Authors:** Zahra Darabi, Seyed Mostafa Seyed Hosseini, Mohammadtaghi Sarebanhassanabadi, Sara Jambarsang, Mohammad Yahya Vahidi Mehrjardi, Mahdieh Hosseinzadeh, Sara Beigrezaei, Azam Ahmadi Vasmehjani, Marzieh Taftian, Vahid Arabi, Maryam Motallaei, Faezeh Golvardi Yazdi, Amin Salehi-Abargouei, Azadeh Nadjarzadeh

**Affiliations:** 1 Department of Nutrition, School of Public Health, Shahid Sadoughi University of Medical Sciences, Yazd, Iran; 2 Research Center for Food Hygiene and Safety, School of Public Health, Shahid Sadoughi University of Medical Sciences, Yazd, Iran; 3 Yazd Cardiovascular Research Center, Non-communicable Diseases Research Institute, Shahid Sadoughi University of Medical Sciences, Yazd, Iran; 4 Departments of Biostatistics and Epidemiology, Center for Healthcare Data Modeling, School of Public Health, Shahid Sadoughi University of Medical Sciences, Yazd, Iran; 5 Department of Genetic, Shahid Sadoughi University of Medical Sciences, Yazd, Iran; 6 Julius center for health sciences and primary car, University of Medical Center Utrecht, Utrecht, Netherlands; 7 Department of Nursing, School of Nursing and Midwifery, Shahid Sadoughi University of Medical Sciences and Health Services, Yazd, Iran

**Keywords:** Genetics, Medical research

## Abstract

Coronary artery disease (CAD) is one of the most important cardiovascular diseases. Lifestyle and genetic factors play important roles in the development of CAD. The aim of the study is to examine the interaction of dietary patterns and genes on the likelihood of abnormal lipid profile and coronary artery stenosis in Iranians undergoing coronary angiography. This cross-sectional study was performed on 440 patients who underwent coronary angiography. The factor analysis method was used to extract dietary patterns. Commercial kits have been used to assess biochemical parameters. The detection of the rs28362491 genotype was carried out by the method of restriction fragment length polymorphism. Traditional (TDP) and western dietary pattern (WDP) were extracted. We observed an interaction of adherence to TDP and rs28362491 on the odds of having a high Gensini score. These interactions indicated that higher adherence to TDP was associated with higher odds of having a high Gensini score for patients with DD genotype than for those with II genotype. (OR 2.33, 95%CI 1.00–5.44; *P* = 0.05). These interactions remained statistically significant even after confounder variables. We observed an interaction between higher adherence to TDP and rs28362491 variants on the odds of high low-density lipoprotein cholesterol levels (*P* = 0.04) in the unadjusted model. We found a significant interaction of this polymorphism and higher adherence to WDP on the odds of having a high Gensini score in the unadjusted model (*P* = 0.04). This study provides a basis for future research on NF-KB1 gene and diet interaction. More large-scale longitudinal studies are needed to validate these findings.

## Introduction

CAD is the most common chronic disease worldwide and it remains the leading global cause of mortality and morbidity^1,2^. CAD is one of the major cardiovascular diseases. It is manifested by stable angina, unstable angina, myocardial infarction, or sudden cardiac death^3^. It results from atherosclerotic blockages in the coronary arteries. Atherosclerosis is a chronic inflammatory disease. It starts when lipoproteins enter and stay in the arterial wall, triggering an inflammatory response that accelerates atherosclerosis^4^.

Genetic, physiological, and biochemical factors contribute to atherosclerotic vascular disease. The main risk factors are obesity, type 2 diabetes mellitus, hypertension, high serum levels of triglyceride, LDL_C, apoprotein, and inflammatory markers, and low serum levels of high-density lipoprotein cholesterol (HDL-C)^5^.

Some studies suggest that diet can directly affect atherosclerosis and also indirectly influence traditional risk factors, such as plasma lipids, blood pressure, and plasma glucose^6^. Evangelos et al. reported WDP was directly associated with the severity of coronary artery lesions in patients with stable coronary artery disease^7^. Several observational studies indicated that vegetarian dietary patterns reduce mortality and the risk of coronary heart disease^8,9^.

Major genomic studies indicated that genetic factors can increase the odds of CAD by 1.1–1.3 times^10^. Some genes that are involved in inflammation can change risk of CAD, according to some new studies^11,12^. Nuclear factor-KB (NF-KB) is a ubiquitous pleiotropic family of transcription factors. NF-KB1 proteins have 5 different types. They are combinations of parts with names p50/p105, p52/p100, p65, RelB, and c-Rel^13^. P50/p105 (NF-KB1) can cause inflammation and increases gene expression such as Tumor necrosis factor-a (TNF-a) and interleukin-12 (IL-12)^14^. NF-KB1 gene is on a part of chromosome 4 and makes two proteins, one big (p105) and one small (p50). Rs28362491 is located in − 94 bp of NF-KB1 promoter. This polymorphism has four bases ATTG insertion/deletion mutants (− 94ins/del ATTG) rs28362491^13^. There are three kinds of genes based on the change: II means both genes have ATTG, DD means both genes lack ATTG, and ID means one gene has ATTG and the other doesn't^15^. The gene can be less active and make less NF-KB1 if ATTG is not there^16^. One study reported that people with DD genotype, who don’t have ATTG, had a higher chance of getting CAD^17^. Results of meta-analysis study have indicated that subjects with the DD genotype had a 1.37-time risk of CAD compared to subjects with the II genotype^18^.

In this study, we aimed to identify the association between dietary patterns and odds of abnormality of lipid profile and coronary artery stenosis as well as to investigate whether there is an interaction of dietary patterns and genes on the odds of abnormal lipid profile and coronary artery stenosis among the Iranian undergoing coronary angiography.

## Method

We conducted this study on 440 patients who underwent coronary angiography. This study was conducted in 2021–2020. This hospital is a central place that takes care of people with heart issues. Adults aged between 35 and 75 years undergoing coronary angiography were recruited for the study. We did not include people who had certain health problems, such as autoimmune diseases, cancer, or liver or kidney failure. We also did not include people who had previous heart procedures, such as Percutaneous coronary intervention (PCI) and coronary artery bypass grafting (CABG), or women who were pregnant or breastfeeding. People who were very overweight (Body mass index (BMI) over 40 kg/m^2^) were also excluded. All participants provided informed consent. We followed the ethical rules of the Declaration of Helsinki. The Ethics Committee of Shahid Sadoughi University of Medical Sciences, Yazd, Iran approved our study (Code number: IR.SSU.SPH.REC.1400.108).

### Extraction DNA and genotyping

Genomic DNA was purified from peripheral vein blood leukocytes using a whole blood genome extraction kit (SIMBIOLAB, IRAN) across the manufacturer’s instructions which were stored at − 20 °C for future analysis. We used RFLP method to detect of genotype the rs28362491 (− 94ATTGins/del) polymorphism of the NF-KB1 gene. The primers for rs28362491 (− 94ATTGins/del) polymorphism of the NF-KB1 gene was adopted from the prior studies^19^. The primers for amplification of the DNA fragment are as follow: 5′-TGGGCACAA GTCGTTTATGA-3′ and 5′-CTGGAGCCGGTAGGGAAG-3′. The protocol of undigested Polymerase chain reaction (PCR) was an initial temperature of 94 °C (5 min) followed by 35 cycles of amplification (30 s at 94 °C, 45 s at 59 °C, and extension for 1 min at 72 °C). The final extension step was conducted for 2-min at 72 °C. Then, undigested PCR products were digested using 1 U of PfIMI restriction enzyme (Van91I, 10 U/μL, Fermentas International Inc, Canada). This mix was incubated overnight at 37 °C. After incubation, the products run on electrophoresis for 45 min at 90 V in 2% agarose gel. Three DNA fragments indicate different lengths: homozygous II (2 bands: 45 and 240 bp), heterozygous DI (3 bands: 285 and 240 and 45 bp), and homozygous DD (1 band: 285 bp).

### Biochemical assessment

We took blood samples from the patients in the hospital after they did not eat anything for a night. We used special tubes with Ethylen ediaminetetraacetic acid (EDTA) to collect the blood and then samples were centrifuged at 5000 rpm for 5 min to separate plasma from blood cells. Serums were store at 80 °C for further analysis. Levels of glucose, total cholesterol, Triglyceride (TG), LDL-C and, HDL-C (Biorex Fars, Iran) were measured by commercial kits. The abnormal serum HDL-C was defined as follows: levels of HDL-C ˂ 40 mg/dl for males and ˂ 50 mg/dl for females^20^. Total cholesterol above 200 mg/dL and LDL-C above 100 mg/dL were considered as abnormal levels^21^. Hypertriglyceridemia is defined as the level of TG being higher than 150 mg/dl^20^.

### Assessment of syntax and Gensini score

Based on the American Heart Association categories, the vessel of coronary is divided into 16 segments. The Syntax score is calculated across the points determined for each individual lesion diagnosis in the coronary vessels with higher than 50% diameter narrowing in vessels > 1.5-mm diameter. Based on the presence of disease, a score of 1 or 2 is given to each section. Then, these scores are weighted based on the type of vessel. Lower Syntax scores are defined as ˂ 22^22^. We get this number by doing these steps: first, we give a score of 0, 1, 2, 4, 8, 16, or 32 to each blockage depending on how much it narrows the vessel. Then, we multiply that score by another number that shows how important the vessel is for the blood flow in the heart. Finally, we add up all the scores for each blockage in a person to get their total Gensini score^23^. Lower Gensini scores are defined as ˂ 20^24^.

### Assessment of anthropometric

Weight was measured by Omron BF511 portable digital scale to the nearest 0.1 kg. Height was measured by trained investigators to the nearest 0.1 cm across the standard protocol. BMI was calculated follow as: dividing the body weight (kg) by the square of height (m).

### Assessment of dietary intake

For the assessment of dietary intake food frequency questionnaire (FFQ) including 178 food items was used. This questionnaire was a modified version of a previously validated 168-item FFQ. Ten questions were added to the previous questionnaire that related to the intake of Yazd-specific food items. The validity and reliability of this questionnaire were assessed in previous studies^25^. Participants have been asked to answer the questionnaire by trained interviewers. The participants had to say how much and how often they ate each food per month, week, or day. We used a guide of household scales to change the amount of food they said to grams^26^.

### Assessment of other variables

We also asked participants about other factors, such as their age, smoking habits, health problems, medicines they took, and economic status. We trained some interviewer to ask these questions to the participants. Levels of physical activity were assessed by International Physical Activity Questionnaire (IPAQ). The validity and reliability of this questionnaire were assessed in a previous study^27^. Levels of physical activity were assumed based on metabolic equivalent task (MET) minutes per week and categorized into low, medium, and high levels of physical activity.

### Statistical analysis

Principal component analysis with the factors rotated by orthogonal transformation was used for determining the dietary pattern. We determined the number of factors to retain based on the following criteria: the interpretability of the factors, the scree test, and the components with an eigenvalue greater than 1^28^. We also examined the correlation matrix between the 34 food groups using the Bartlett test of sphericity and the Kaiser–Meyer–Olkin (KMO) test. The extracted dietary patterns were labeled across food groups that had a factor loading greater than 0.3^29^. We calculated the score for each dietary pattern for every patient by summing the standardized amount of the food groups multiplied by their scoring coefficient. These scores measured how closely each participant followed the identified pattern. Participants were divided into low and high adherence groups based on the median of each dietary pattern score. We performed one-way ANOVA and chi-square tests to detect significant differences in genotype variants for quantitative and qualitative variables, respectively. We used independent sample t-test to compare the continuous variables between low and high adherence groups for each dietary pattern. We conducted stratified analyses based on adherence to dietary patterns. We applied the multivariable logistic regression method to estimate the odds ratios (OR) of the rs28362491 variants with abnormal lipid profiles and severity of atherosclerosis in vessels.

To examine the interaction between the NF-KB1 rs28362491 polymorphisms and adherence to the dietary patterns, or other dietary variables, on abnormal lipid profiles and severity of atherosclerosis, we employed the multivariate regression models. We performed statistical analysis using SPSS 23.0.

### Ethical approval

This study was approved by the Ethics Committee of Shahid Sadoughi University of Medical Sciences, Yazd, Iran.

### Consent to participate and publish

All participants were consented written informed for entering and publication of study results.

## Results

### Study population characteristics

We conducted the present study on 440 patients who underwent coronary angiography with a mean age of 56.9 years. Factor analyses identified two main dietary patterns. Table 1 shows the factor-loading matrices for the two dietary patterns.

**Table 1:** Factor loading matrix for the two major dietary patterns.

Food group	Traditional dietary pattern	Western dietary pattern
Pickles	0.609	
Vegetables	0.577	
Fruits	0.533	
Sugar	0.507	
Red meats	0.455	
Butter	0.448	
Fruit juice	0.433	
Broth	0.418	
Animal fats	0.388	
Nuts	0.373	
Dairy	0.373	
Tea	0.364	
Legumes	0.323	0.341
Olive oil		
Industrial fruit juice		0.544
Sweetened beverages		0.531
Coffee		0.471
Sweet desserts		0.463
Eggs		0.461
Refined grains		0.446
Fish		0.392
Vegetable oils		0.392
Processed meats		0.384
Poultry		0.375
Mayonnaise and fried foods		0.309
Variance explained (%)	8.99	8.04

Factor loadings higher than ± 0.3 are shown.

Factor 1, which had a high intake of pickles, vegetables, fruits, sugar, red meats, butter, fruit juice, broth, animal fat, nuts, dairy products, tea and legumes, was named the TDP. Factor 2, which had a high intake of industrial fruit juice, sweetened beverages, coffee, sweet desserts, eggs, refined grains, fish, vegetable oil, processed meats, poultry, legumes, mayonnaise and fried foods, was named the WDP.

Table 2 shows the general characteristics and dietary intake of study participants by rs28362491 genotypes and adherence to WDP and TDP. BMI, gender, smoking, physical activity and drug use did not differ among genotype variants. Patients with the highest adherence to TDP were more physically active than those with the lowest adherence. Medication use, smoking and gender differed significantly between adherence to TDP and WDP. Intake of energy, protein, carbohydrate, polyunsaturated fatty acid (PUFA), monounsaturated fatty acid (MUFA), saturated fatty acid (SFA) cholesterol, vitamin C, E, B12 and folate did not vary significantly among genotypes. Participants with the highest adherence to TDP and WDP had higher intakes of energy, protein, carbohydrate, PUFA, MUFA, SFA, cholesterol, vitamin C, E and B12 than those with the lowest adherence.

**Table 2:** Characteristics and dietary intake of study participants according to rs28362491 genotypes and adherence to WDP and TDP.

Variables	Type of genotype			WDP				TDP		
	DD	DI	II	P**	Low	High	P**	Low	High	P**
Age (years)*	59.40 ± 8.59	55.98 ± 9.38	57.41 ± 9.87	0.02	58.78 ± 8.80	55.17 ± 9.74	< 0.001	28.12 ± 4.48	26.66 ± 3.96	0.69
BMI (kg/m^2^)*	27.08 ± 4.79	27.80 ± 4.19	26.83 ± 4.10	0.10	27.77 ± 4.43	27.01 ± 4.11	0.07	56.79 ± 9.71	57.15 ± 9.20	< 0.01
Gender, male, n (%)	51 (66.2)	148 (63)	74 (58.7)	0.53	104 (47.3)	170 (77.6)		109 (49.8)	165 (75)	< 0.001
Smoking, n (%)										
Non smoker	49 (63.6)	153 (65.7)	81 (64.3)	0.37	169 (76.8)	114 (52.8)	< 0.001	162 (74.3)	121 (55.5)	< 0.001
Former smoker	6 (7.8)	8 (3.4)	3. (2.4)		7 (3.2)	10 (4.6)		8 (3.7)	9 (4.1)	
Current smoker	22 (28.6)	72 (30.9)	42 (33.3)		44 (20)	92 (42.6)		48 (22)	88 (40.4)	
Physical activity, n (%)										
Low	24 (31.6)	82 (35.5)	38 (30.6)	0.91	83 (38.1)	61 (28.6)	0.10	85 (39)	59 (27.7)	0.04
Moderate	26 (34.2)	74 (32)	43 (34.7)		69 (31.7)	74 (34.7)		68(31.2)	75 (35.2)	
High	26 (34.2)	75 (32.5)	43 (34.7)		66 (30.3)	78 (36.6)		65 (29.8)	79 (37.1)	
Medication use, yes, n (%)										
Statins	28 (36.4)	89 (37.9)	39 (30.7)	0.39	92 (41.8)	64 (29.2)	< 0.001	96 (43.8)	60 (27.3)	< 0.001
Antidiabetics	22 (28.6)	82 (34.9)	41 (32.2)	0.57	85 (38.6)	60 (27.4)	0.01	84 (38.4)	135 (61.6)	0.01
Dietary intake										
Energy intake (kcal)*	2622.21 ± 1274.69	2704.0) ± 1202.18	2738.01 ± 120.87	0.80	2010.26 ± 744.03	3402.99 ± 1207.39	< 0.001	2153.75 ± 1025.51	3259.50 ± 1148.85	< 0.001
Protein(gr)*	92.98 ± 45.47	102.59 ± 54.03	103.95 ± 54.12	0.30	71.65 ± 30.09	131.48 ± 53.71	< 0.001	82.49 ± 46.29	120.08 ± 52.13	< 0.001
Carbohydrate (gr)*	410.49 ± 203.27	407.90 ± 185.72	417.99 ± 198.26	0.89	316.14 ± 129.65	508.17 ± 197.24	< 0.001	327.95 ± 165.56	494.60 ± 180.74	< 0.001
Fat (gr)*	72.51 ± 45.41	76.90 ± 44.10	78.63 ± 39.49	0.61	55.53 ± 28.06	98.11 ± 44.96	< 0.001	56.86 ± 28.84	96.39 ± 45.74	< 0.001
Saturated fatty acid (gr)*	22.08 ± 16.12	22.77 ± 14.27	23.89 ± 12.7	0.64	17.84 ± 11.93	28.20 ± 14.41	< 0.001	16.76 ± 8.86	29.18 ± 15.73	< 0.001
PUFA( gr)*	17.09 ± 11.43	19.00 ± 14.04	18.72 ± 11.14	0.52	12.68 ± 7.53	24.59 ± 14.22	< 0.001	13.65 ± 7.84	23.51 ± 14.79	< 0.001
MUFA (gr)*	17.09 ± 11.4	19.00 ± 14.04	18.72 ± 11.14	0.50	16.59 ± 8.11	29.84 ± 13.12	< 0.001	17.67 ± 8.91	28.64 ± 13.61	< 0.001
Cholesterol (mg)*	415.83 ± 295.56	446.00 ± 260.69	461.90 ± 283.18	0.50	332.44 ± 203.02	560.19 ± 288.16	< 0.001	368.96 ± 205.56	521.57 ± 309.78	< 0.001
Vitamin C (mg)*	256.55 ± 163.31	249.61 ± 175.76	258.93 ± 202.82	0.88	203.68 ± 152.46	304.30 ± 194.70	< 0.001	170.26 ± 110.97	336.80 ± 199.64	< 0.001
Vitamin E (mg)*	17.09 ± 11.43	13.89 ± 9.79	13.53 ± 8.16	0.84	9.48 ± 5.44	17.89 ± 10.16	< 0.001	10.12 ± 5.95	17.17 ± 10.35	< 0.001
Folate (mcg)*	16.06 ± 13.66	21.06 ± 34.81	17.43 ± 15.25	0.70	355.88 ± 210.90	571.85 ± 251.26	< 0.001	340.25 ± 161.38	585.50 ± 273.14	< 0.001
Vitamin B12 (mcg)*	3.43 ± 1.90	4.22 ± 3.85	4.40 ± 3.18	0.12	2.90 ± 1.71	5.38 ± 4.16	< 0.001	3.52 ± 3.70	4.75 ± 2.95	< 0.001

The degree of adherence to dietary patterns is categorized into two categories, “high” adherence and “low” adherence, based on the median intake.

*BMI* body mass index, *SFA* saturated fatty acid, *MUFA* monounsaturated fatty acid, *PUFA* polyunsaturated fatty acid, *WDP* western dietary pattern, *TDP* traditional dietary pattern.

*Data presented as mean ± SD.

**Obtained from Chi-squared test and independent t-test for categorical and continuous variables respectively.

### Comparison of abnormal lipid profile and score of coronary artery stenosis between rs28362491 genotypes and adherence to dietary patterns

Table 3 shows the comparison of the Gensini score, Syntax score and lipid profile by rs28362491 genotypes and adherence to WDP and TDP. The number of patients with abnormal HDL, LDL, TG and high Gensini and Syntax scores did not vary significantly among genotype variants and adherence to dietary patterns. The number of patients with high cholesterol differed significantly among genotypes. The patients with the DI genotype had higher total cholesterol levels than those with the DD and II genotypes.

**Table 3:** Comparison of the Gensini score, Syntax score and lipid profile according to rs28362491 genotypes and adherence to WDP and TDP.

rs28362491genotypes					WDP				TDP	
	II	DI	DD	P*	Low	High	P*	Low	High	P*
High Gensini score, n (%)	38 (52.1)	112 (49.8)	56 (46.3)	0.71	102 (48.8)	104 (49.3)	0.92	103 (47.9)	103 (50.2)	0.63
High syntax score, n (%)	15 (20.5)	40 (17.8)	22 (18.2)	0.86	37 (17.7)	40 (19)	0.74	36 (16.7)	41 (20)	0.38
High triglyceride, n (%)	27 (40.3)	84 (36.8)	42 (37.8)	0.87	86 (42.6)	68 (33.2)	0.05	74 (35.7)	80 (40)	0.37
High cholesterol, n (%)	19 (28.4)^a^	93 (41)^b^	33 (29.7)^a^	0.04	66 (32.7)	80 (39.2)	0.17	82 (39.8)	64 (32)	0.10
High LDL-C, n (%)	26 (39.4)	99 (43.6)	50 (45)	0.75	90 (44.8)	86 (42.2)	0.59	99 (48.1)	77 (38.7)	0.05
Low HDL-C, n (%)	25 (37.9)	76 (33.2)	37 (33.6)	0.77	76 (37.6)	62 (30.4)	0.12	74 (35.9)	64 (32)	0.40

The degree of adherence to dietary pattern is categorized into two categories, “high” adherence and “low” adherence, based on the median intake.

*HDL-c* high density lipoprotein cholesterol, *LDL-c* low density lipoprotein cholesterol, *WDP* western dietary pattern, *TDP* traditional dietary pattern.

*Obtained from Chi-squared test.

^ab^Bonferroni correction.

### Interaction between the NF-KB1 rs9939609 and dietary patterns with abnormal lipid profile and coronary artery stenosis score

This study found a significant interaction between adherence to the TDP and rs28362491 on the odds of high Gensini score (Table 4). Based on these interactions, when adherence to the TDP was high, patients with the DD genotype had higher odds of high Gensini score than those with the II genotype (OR 2.33, 95%CI 1.00–5.44; *P* = 0.05). However, when adherence to the TDP was low, there was no association of these polymorphisms with odds of high Gensini score (OR 0.70, 95%CI 0.30–1.63; *P* = 0.41). These interactions remained statistically significant even after adjustment for age, gender, energy intake, physical activity, smoking, economic status, medication use, and BMI (*P* interaction = 0.02). There was an interaction between adherence to TDP and variants of rs28362492 on odds of high LDL levels (*P* interaction = 0.04) but this interaction was not statistically significant after adjustment for age, gender, energy intake, physical activity, smoking, economic status, medication use, BMI.

**Table 4:** Association between the Gensini score, syntax score, lipid profile and rs28362491 genotypes in difference adherence to TDP.

	Model 1		P* interaction gene × TDP	Model 2		P* interaction gene × TDP
	Adherence to the TDP			Adherence to the T		
	Low	High		Low	High	
	OR CI	OR CI		OR CI	OR CI	
High Gensini score						
II	1.00 (references)	1.00 (references)	0.04	1.00 (references)	1.00 (references)	0.02
DI	1.25 (0.66–2.36)	1.03 (0.55–1.93)		1.02 (0.48–2.20)	0.96 (0.45–2.05)	
DD	0.70 (0.30–1.63)	2.33 (1.00–5.44)		0.42 (0.15–1.16)	2.17 (0.79–5.99)	
P**	0.41	0.05		0.09	0.13	
High syntax score						
II	1.00 (references)	1.00 (references)	0.21	1.00 (references)	1.00 (references)	0.10
DI	0.68 (0.30–1.52)	1.39 (0.60–3.18)		0.67 (0.26–1.72)	1.33 (0.52–3.38)	
DD	0.57 (0.18–1.78)	2.11 (0.78–5.71)		0.55 (0.15–1.94)	2.27 (0.75–6.84)	
P**	0.33	0.13		0.35	0.14	
High triglyceride						
II	1.00 (references)	1.00 (references)	0.76	1.00 (references)	1.00 (references)	0.67
DI	0.94 (0.48–1.86)	0.98 (0.51–1.88)		1.01 (0.44–2.31)	0.75 (0.34–1.68)	
DD	0.90 (0.36–2.22)	1.38 (0.57–3.29)		1.07 (0.36–3.20)	1.37 (0.49–3.82)	
P**	0.83	0.46		0.89	0.54	
High LDL-C						
II	1.00 (references)	1.00 (references)	0.04	1.00 (references)	1.00 (references)	0.25
DI	1.66 (0.85–3.21)	0.51 (0.27–0.98)		1.34 (0.63–2.82)	0.60 (0.29–1.24)	
DD	1.03 (0.42–2.49)	0.62 (0.25–1.49)		1.01 (0.38–2.68)	0.61 (0.23–1.62)	
P**	0.94	0.28		0.97	0.32	
Low HDL-C						
II	1.00 (references)	1.00 (references)	0.40	1.00 (references)	1.00 (references)	0.76
DI	0.80 (0.40–1.58)	1.16 (0.58–2.33)		1.09 (0.49–2.39)	1.00 (0.45–2.24)	
DD	0.96 (0.39–2.34)	1.48 (0.59–3.68)		1.38 (0.49–3.83)	1.15 (0.39–2.35)	
P**	0.92	0.32		0.53	0.79	
High cholesterol						
II	1.00 (references)	1.00 (references)	0.17	1.00 (references)	1.00 (references)	0.35
DI	2.53(1.24–5.16)	1.03(0.52–2.04)		2.67(1.15–6.18)	1.08(0.49–2.38)	
DD	1.08(0.41–2.82)	0.82(0.32–2.11)		1.18(0.40–3.53)	0.90(0.29–2.71)	
P**	0.86	0.68		0.75	0.85	

The degree of adherence to dietary pattern is categorized into two categories, “high” adherence and “low” adherence, based on the median intake.

Model 1: crude model.

Model 2: Adjusted for age, gender, energy intake, physical activity, smoking, economic status, medication use, BMI.

*HDL-c* high density lipoprotein cholesterol, *LDL-c* low density lipoprotein cholesterol, *TDP* traditional dietary pattern.

P* value for the interaction between adherence to the traditional dietary pattern and the rs28362491 genotypes in the logistic regression model.

P** DD genotype compared to II genotype.

There was a significant interaction effect of the NF-KB1rs28362491 variant and the WDP on the risk of severe coronary artery disease in the crude model (*P* = 0.04), but not in the adjusted model (Table 5).

**Table 5:** Association between the Gensini score, syntax score, lipid profile and rs28362491 genotypes in difference adherence to WDP.

	Model 1		P* interaction gene × WDP	Model 2		P* interaction gene × WDP
	Adherence to the WDP			Adherence to the WDP		
	Low	High		Low	High	
	OR CI	OR CI		OR CI	OR CI	
High Gensini score						
II	1.00 (references)	1.00 (references)	0.04	1.00 (references)	1.00 (references)	0.08
DI	1.68 (0.90–3.14)	0.78 (0.41–1.48)		1.56 (0.71–3.43)	0.77 (0.36–1.64)	
DD	0.99 (0.44–2.24)	1.63 (0.69–3.86)		0.75 (0.27–2.02)	1.50 (0.55–4.07)	
P**	0.99	0.26		0.57	0.42	
High syntax score						
II	1.00 (references)	1.00 (references)	0.13	1.00 (references)	1.00 (references)	0.35
DI	1.81 (0.75–4.35)	0.55 (0.25–1.21)		1.56 (0.59–4.11)	0.62 (0.25–1.54)	
DD	1.58 (0.52–4.77)	0.91 (0.33–2.45)		1.35 (0.41–4.44)	0.90 (0.30–2.72)	
P**	0.41	0.85		0.61	0.86	
High triglyceride						
II	1.00 (references)	1.00 (references)	0.09	1.00 (references)	1.00 (references)	0.18
DI	1.60 (0.82–3.11)	0.57 (0.29–1.12)		1.55 (0.70–3.46)	0.47 (0.20–1.10)	
DD	1.61 (0.69–3.75	0.70 (0.27–1.79)		1.72 (0.63–4.64)	0.93 (0.30–2.90)	
P**	0.26	0.46		0.28	0.91	
High LDL-C						
II	1.00 (references)	1.00 (references)	0.17	1.00 (references)	1.00 (references)	0.09
DI	1.03 (0.54–1.97)	0.85 (0.44–1.63)		1.22 (0.57–2.59)	0.72 (0.35–1.48)	
DD	1.32 (0.57–3.04)	0.40 (0.15–1.07)		1.59 (0.61–4.13)	0.33 (0.11–0.96)	
P**	0.51	0.07		0.33	0.04	
Low HDL-C						
II	1.00 (references)	1.00 (references)	0.42	1.00 (references)	1.00 (references)	0.34
DI	0.73 (0.38–1.42)	1.42 (0.68–2.97)		0.70 (0.31–1.56)	1.76 (0.76–4.11)	
DD	1.01 (0.43–2.35)	1.50 (0.56–4.01)		1.11 (0.40–3.06)	1.56 (0.51–4.75)	
P**	0.97	0.42		0.83	0.42	
High cholesterol						
II	1.00 (references)	1.00 (references)	0.12	1.00 (references)	1.00 (references)	0.12
DI	2.47 (1.17–5.21)	1.12 (0.58–2.17)		3.64 (1.43–9.21)	1.20 (0.56–2.56)	
DD	1.84 (0.72–4.69)	0.46 (0.16–1.27)		2.28 (0.75–6.27)	0.46 (0.14–1.47)	
P**	0.20	0.13		0.14	0.19	

The degree of adherence to dietary pattern is categorized into two categories, “high” adherence and “low” adherence, based on the median intake.

Model 1: crude model.

Model 2: Adjusted for age, gender, energy intake, physical activity, smoking, economic status, medication use, BMI.

*HDL-c* high density lipoprotein cholesterol, *LDL-c* low density lipoprotein cholesterol, *TDP* traditional dietary pattern.

P* value for the interaction between adherence to the traditional dietary pattern and the rs28362491 genotypes in the logistic regression model.

P** DD genotype compared to II genotype.

## Discussion

To our best knowledge, this was the first study to explore the gene-diet interaction on coronary artery stenosis and its risk factors. We found no association of the rs28362491 variant with lipid profile or coronary artery disease severity. Previous results about the association between genotype variants of this polymorphism with CAD and risk factors were not consistent. Lai et al. reported that individuals with the NF-KB1-94 DD genotype had 1.4-time increase in odds of CAD compared to subjects with the II genotype in male patients^30^.

Another study has reported that healthy subjects had a higher D allele frequency of rs28362481 compared to the myocardial infarction patients. Having the ins/del ATTG type of the ins/del ATTG NF-KB1gene might protect against heart attacks^31^. One study has reported that acute coronary syndrome patients with the DD genotype had higher stenosis of the coronary artery compared to subjects with ID and II genotypes^32^.

The Gene of NF-KB1 has an important role in the regulation of transcription of some 200 target genes and inflammatory responses^30^. Subunits of this gen determined the inflammatory responses. The p50 subunit can reduce inflammation by lowering the production of pro-inflammatory cytokines such as TNF-α and IL-12^33^. The NF-KB1 gene also has another part called P105. NF-KB1 -94delATTG allele in the promoter region of the NF-KB1 gene impairs transcription factor binding site and leads to lower activity of promoter transcriptional and less p50 biosynthesis^30^. It has been well known that inflammation plays an important role in the severity of coronary artery disease and the development of CAD^34^.

However, there is no association between genotype variants of rs28362491 and high Gensini score in this study, but there was a significant association among participants with high adherence to TDP and WDP. Patients with the DD genotype of the gene and high adherence to TDP had a higher odds of severe steatosis coronary (high Gensini score). Association between adherence to WDP and variants of rs28362491 with Gensini score was disappear after adjustment for confounder variable. In our study, there was an interaction between variants of rs28362491 and adherence to TDP on LDL-C in the crude model. Subjects with DD genotype who adhered more to TDP had higher odds of elevated LDL-C levels.

Previous studies investigated the association of dietary patterns with CAD and its risk factors. The cohort study results indicate that the TDP, which consists of high intakes of whole grains, vegetables, legumes, white and red meats, hydrogenated fats, fresh and dried fruits, high-fat and low-fat dairy products, nuts and seeds, is not associated with increased cardiovascular disease risk in the Iranian population^35^. Another study found a positive association between higher adherence to TDP and CAD risk factors. TDP was characterized by high consumption of red meat, grains, egg, organ meat, solid oil, frying oil, butter, sugar, dough, and tea^36^. In our study, the TDP, which included red meat, animal fats, broth, and butter, was associated with high PUFA intake and low omega-3 to omega-6 ratio among patients who adhered more to this pattern. Some studies suggested that n − 3 fatty acids intake reduced the expression of genes involved in inflammatory and atherogenic pathways, such as NF-KB1 signaling^37^. Omega 6 PUFA metabolites, such as prostaglandin 2 (PG2), enhanced the production of inflammatory cytokines and NF-KB1^38^. A previous study examined the interaction of PUFA intake and this polymorphism on lipid profile. The study results showed that higher energy intake from PUFA was linked to higher HDL-C levels in individuals with the II genotype, while the opposite was true for those with the DD genotype^39^.

This study had some limitations. First, this was a cross-sectional study, so causal associations could not be inferred. Second, the FFQ was used for dietary assessment, which may have memory bias. Third, this study was conducted on Iranian patients undergoing angiography, and the results may not be generalizable due to racial and regional differences. This study also had several strengths. This was the first study to investigate the interaction between NF-KB1 rs28362491 polymorphism and dietary patterns on cardiovascular risk factors and severity of coronary steatosis. The results of these assessments could play a role in providing personalized nutritional recommendations to manage and modify the risk factors of CAD.

## Conclusion

Subjects with the DD genotype of rs28362491 may be more sensitive to traditional dietary pattern. The results of this study could serve as a basis for future research on NF-KB1 gene and diet interaction. Large prospective studies are needed to validate these results.

## Data Availability

The data and materials of the current study is available from the corresponding author, Azadeh Najarzadeh, on reasonable request.
